# Learning from the Experts: Stimulating Student Engagement in Small-group Active Learning

**DOI:** 10.5334/pme.1245

**Published:** 2024-04-15

**Authors:** Jan Willem Grijpma, Siema Ramdas, Louti Broeksma, Martijn Meeter, Rashmi A. Kusurkar, Anne de la Croix

**Affiliations:** 1 Research in Education, Amsterdam UMC, Amsterdam, the Netherlands; 2 Centre for Teaching and Learning, Vrije Universiteit Amsterdam, Amsterdam, the Netherlands; 3 Research in Education, Amsterdam UMC, location Vrije Universiteit Amsterdam, Amsterdam, the Netherlands; 4 Vrije Universiteit Amsterdam, Amsterdam, the Netherlands

## Abstract

**Introduction:**

Engaging students in small-group active learning methods is essential for their development. Yet, medical teachers frequently face difficulties in stimulating this engagement, resulting in students remaining passive or detached from the learning process. The aim of this study was to uncover ways in which expert medical teachers, proficient at cultivating high levels of student engagement, stimulate such engagement. This knowledge might inform faculty development initiatives, so that medical teachers can be better equipped to teach in a way that engages students.

**Methods:**

We conducted an interview study using a constructivist grounded theory approach, integrating elements from appreciative inquiry. The eleven participants were qualified medical teachers who repeatedly received high scores on student engagement. Each interview was transcribed, coded, and analyzed using constant comparison until theoretical saturation was achieved.

**Results:**

We constructed a grounded theory of expert teaching practice, describing student engagement as an integrated process consisting of three components: 1) aiming for a supportive learning environment; 2) employing a personal educational approach; and 3) facilitating the active learning process.

**Discussion:**

This study uncovered that there are multiple ways to stimulate high levels of student engagement. Although there was consensus on the importance of a supportive learning environment and the ability to facilitate the active learning process, participants recognized the contextual nature of student engagement and took on a reflective mindset to adapt strategies to their specific situations. These findings highlight the need for faculty development initiatives to adopt a comprehensive, context-sensitive approach that considers the complexity of student engagement.

## Introduction

Small-group active learning methods, which involve interactive student-centered activities, can improve medical students’ knowledge, skills, and personal and professional competencies when they stimulate student engagement [1,2]. Student engagement, more recently also called learning engagement [3], has been conceptualized as the cognitive, emotional, and behavioral aspects of students’ involvement in learning activities [4]. When students engage in interactive and constructive ways with the subject matter, their learning increases [5]. In reviews describing the specific conditions necessary for student engagement in active learning methods, ‘the teacher’ is mentioned consistently as a determinant of success [6,7,8,9,10,11]. Teachers need to be competent in cultivating a learning environment in which students can engage with each other and the subject matter to develop themselves. Students who perceive active learning to be poorly designed or executed by their teacher have been shown to disengage from learning [12,13]. Therefore, for small-group active learning to be successfully implemented and contribute to student development, there is an urgent need for both novice and advanced medical teachers to improve their mastery in student engagement [14,15,16,17].

The challenges that medical teachers encounter when engaging their students seem to arise from various sources. First, teachers themselves have personal views on education, which may or may not align with active learning as an effective strategy, and which can affect how they approach their teaching tasks [15,18,19,20]. Active learning requires specific teaching competencies that not all teachers may have developed [14,15,21]. Second, student beliefs about learning and associated competencies may or may not align with active learning as an effective strategy, which can influence their behavior in class [14,17,22]. Third, pedagogical and didactical issues may affect the conditions necessary for student engagement, such as class size and the amount of time available for learning activities [14,15]. Finally, institutional challenges may limit the time that teachers can spend on teaching and professional development [15,23,24]. When teachers fail to deal with these challenges, they have been shown to revert to more passive (i.e., less effective) ways of teaching [16].

To support medical teachers in engaging their students and dealing with associated challenges, faculty development serves as a critical resource [15,25,26]. Through faculty development initiatives, teachers are instructed in strategies to positively impact student engagement. These initiatives can yield favorable knowledge, attitudes, and behavioral outcomes [21,27,28]. However, these successes are not the end of the story. In practice, medical teachers continue to encounter difficulties in engaging students in their classrooms. Even experienced medical teachers with advanced knowledge, skills, and positive attitudes toward active learning face challenges when trying to implement the lessons learned from training [15]. Thus, despite the effectiveness of faculty development in offering student engagement strategies, there is a need for additional understanding to support medical teachers in engaging their students.

Despite the reported challenges and insufficient support from faculty development, certain medical teachers have successfully implemented active learning and mastered student engagement. These ‘experts’ could possess valuable insights that could advance our understanding of student engagement. Currently, it is not known what these teachers do in their classrooms. In this study, we set out to learn how successful teachers approach their tasks.

### Aim and research question

Our study aimed to construct a theory of student engagement in small-group active learning settings. This theory could inform faculty development initiatives so that medical teachers can be better equipped to teach in ways that engage students. Our guiding research question was: how do expert medical teachers stimulate high levels of student engagement in small-group active learning sessions?

## Methods

### Research design

We conducted an interview-based study to explore how expert teachers stimulate high levels of student engagement. We used a constructivist grounded theory approach, which is a qualitative research methodology that seeks to understand social processes [29]. It employs an inductive approach to theory development, with data collection and analyses occurring simultaneously and iteratively, making use of constant comparison methods [30,31]. We aimed to include participants who could contribute to the richness of the collected data [31]. To enhance transferability, we carefully described the study context [32,33].

Consistent with constructivist epistemology and the methodology, we viewed student engagement as a social construct shaped by experiences and contextual factors. This stance acknowledged our preconceptions and preexisting beliefs, while the constructivist grounded theory approach guarded against being solely determined by them.

We adhered to the GUREGT (Guideline for Reporting and Evaluating Grounded Theory Research Studies) to ensure study quality and rigor in reporting its process and findings [34].

### Study population and setting

We defined expert teachers as individuals: 1) having obtained, or nearing completion of, a formal teaching qualification (nationally recognized, incorporating training in active learning and student engagement); and 2) attainment of a score of at least 4.0 (on a 1–5 scale) from minimum two study groups on student evaluations concerning student engagement.

In constructivist grounded theory, initial and theoretical sampling procedures are used to collect data [29]. The participants, selected through purposive sampling, were eleven expert teachers involved in a tutoring course offered by the Faculty of Medicine at the Vrije Universiteit Amsterdam (Table 1). This course is taught in all three years of the bachelor’s program. Each year, approximately 150 teachers are involved, teaching 154 study groups, comprising a maximum of twelve students each. The course objectives are related to the integration and application of the knowledge, skills, and attitudes gained in lectures, labs, and other courses. Teachers meet with their study groups once or twice per week during a semester for two hours. Sessions involve a variety of learning activities based on patient cases. In years 1 and 2, the course employs a collaborative-case based learning approach in which the teacher’s task is to guide the active learning process, while the students are responsible for learning the content and running the sessions. Teachers are not required to have a medical background. In year 3, the course employs a team-based learning approach, in which the teachers lead the sessions and are actively involved in discussing the content. Therefore, teachers in year 3 are required to have a medical background.

**Table 1:** Participant characteristics.


Average number of study groups taught		12.2 (range 7–24)

Background	Medical	3

	Para- or nonmedical	8

Sex	Female	6

	Male	5


Consistent with the tutoring course design and its teacher population, participants were involved in all three years, bringing medical and other backgrounds to their teaching, as well as varied teaching experience. Constructivist grounded theory studies benefit from a diverse sample, as it enriches the depth and breadth of generated insights [29].

### Data collection and analyses

The interviews were designed using appreciative inquiry elements [35,36,37,38]. Appreciative inquiry is characterized by a focus on ‘what works well’ instead of ‘what is going wrong’, resulting in participants speaking more openly and less defensively [37]. Our questions reflected this method through our focus on participants’ positive teaching experiences (instances of high student engagement) and collaboratively discovering what underlying processes contributed to those experiences. The interviews were semi-structured (See supplementary materials for interview guide). Each interview was audio-recorded and transcribed for analysis.

We collected and analyzed data concurrently, using Atlas.ti version 22 [39], field notes, and memos. Authors JG, SR, LB, and AC held analysis meetings every 2–4 interviews. We established coding practices to facilitate comparison and discussion of findings. To start, we independently engaged in initial coding and identified possible patterns in the data. During the first meeting, we discussed preliminary codes and memos, and modified the interview guide. Focused coding followed, collaboratively refining codes and concepts that gave meaning to and explained larger portions of data. Through constant comparison, we compared new interviews to previous data, identifying contradictions, expansions, and support. We explored interactions between participant characteristics and the research question to identify their potential influence on the findings. Consequently, we could identify categories and themes with increasing specificity and precision, while also explaining links between the categories and themes through theoretical coding. This iterative process was continued until a stable thematic structure developed, visualized through diagrams and storyline procedures [29,31,40].

Theoretical saturation (i.e., additional data likely do not contribute new insights to the developing theory or categories) was employed to determine if the interviews had yielded the data needed to achieve our research aim [29,41]. We achieved saturation after 11 interviews, after which we reached a sufficient and coherent conceptualization without any significant gaps [29,42].

### Reflexivity

The authors have extensive knowledge of active learning through scholarship and their experiences as teachers and students in courses that employed active learning methods. AC, SR, and JG have extensive faculty development experience that might influence their findings, which were checked and discussed throughout with the entire research team. JG taught a teacher qualification course, through which he knew some participants before conducting the interviews. There was no active relationship between them at the time of the interviews. Participants were aware in advance that JG would be the interviewer and had the option to decline participation or request a different interviewer. RK is a teacher in the tutoring course, but not a participant in the study. Her experiences were discussed during team meetings and helped facilitate the conception and execution of this study. AC had experience in the methodology and guided the team through the study.

### Ethics and consent

Ethical approval was obtained from the Ethical Review Board of the Netherlands Association of Medical Education (dossier number 2020.5.1). Before partaking in the interviews, participants received an information letter about the study, which they could read at their convenience. Then, if they agreed to participate, they signed an informed consent form, and the interview was scheduled. The participants did not receive compensation for participating.

## Results

Analysis of the interview data produced an expert theory of engaging students in small-group active learning. We identified three interacting components: 1) aiming for a supportive learning environment; 2) employing a personal educational approach; and 3) facilitating the active learning process. Given our comprehensive analysis of expert teachers’ strategies for engaging students, the results do not detail concrete behaviors, instead offering a synthesized overview of reported practices and interactions between components.

### Aiming for a supportive learning environment

#### Psychological safety

Participants consistently described how student engagement started with providing psychological safety. This meant that students felt secure, appreciated, and had a sense of belonging, enabling them to contribute, show vulnerability, be themselves, and make mistakes without fear of judgment.

Participants felt that psychological safety was essential in an active learning process. Students in the tutoring course were required to ask questions and provide answers even when they were not certain they would be correct, to give and receive feedback, to give presentations, and to experiment with new behaviors in order to develop new skills. To truly engage in such activities, students required this safety.

*I think a safe atmosphere is the most important for engaging students. It is a precondition. If that is not there… If students are not convinced that making mistakes is okay, that they are there to engage in a learning process… Yeah, then you will not get those little gears in their mind spinning, so to say. That is why I think that is the most important. (P1)*

#### Mutual care and commitment

Participants conveyed genuine care for their students’ well-being and development. According to them, this involved understanding their students on an individual level – knowing about personal lives, interests, qualities, and areas for improvement. They also emphasized being a reliable support person during difficult times and striving to create personal learning opportunities that would facilitate their students’ growth. In turn, they said students reciprocated by adopting a caring and constructive attitude toward their peers and the learning process.

*I remember in the time of COVID, students were just withering away. They didn’t like only being at home. And then I said, you know what, let’s go together to the Amsterdam Forest and have a walk. They appreciated that greatly. I remember, and I really liked that, that they said: ‘you know, you really take care of us’. […] And because I took care of them, they also cared for me. In the sense that, they know what I want. And if they feel that I take care of them they will take care of me by, well, doing their best. (P3)*

#### Clear and shared classroom structure

Participants stated that student engagement required the teachers and the students to negotiate agreements and share responsibility in complying with them. When everyone knew what was expected of them, student engagement improved, and the efficiency and effectiveness of the active learning process increased.

*I aim to establish a sort of democratic decision-making process. The choices that are made, the direction we take with the assignments – whatever we do – it should be shared and supported by everyone. This is essential. The idea is that they all endorse what we are doing. They should have the idea: ‘we are here for ourselves and not because it’s required for the course’. (P8)*

### Employing a personal educational approach

#### Teachers’ educational values and competencies

Participants indicated that their approach to engaging students was shaped by their educational values and competencies. These values (beliefs and guiding principles) included student-centered learning, collaboration, responsibility, personal development, and lifelong learning. Each value informed their daily teaching practices in specific ways. For example, one teacher talking about the value of responsibility:

*It is important to me that students do not just sit back and wait for the curriculum to hand them knowledge. No, they need to develop the competencies required to become a doctor. […] They need to take responsibility for their development and regularly assess their progress. […] That is why I communicate to them about their responsibility. Sometimes, I need to sit on my hands and resist the urge to help them, because of course I want to help them and just tell them what to do. But for their development, that is not the most effective approach. So, I literally tell myself: it was a good session when I did not have to do anything. (P1)*

To effectively guide an active learning process that aligns with their educational values, participants acknowledged the need for advanced competencies. They reported developing these competencies over the years through various faculty development initiatives, conducting ‘experiments’ with their study groups, and through their general experiences as teachers. These activities, in turn, developed their sense of self-efficacy and autonomy, which resulted in being comfortable with their approach to the course in accordance with their values and competencies.

#### Knowledge and beliefs about students

Participants described an awareness of students entering their study groups with specific learning experiences and expectations, as well as personal qualities and needs. As participants learned about these qualities and needs, they could use that information to personalize the active learning process and stimulate engagement at the same time.

*[when starting a learning activity] I do not demand students to speak in a certain order or give them turns. I try very much to steer on what I know of a student: ‘so you told me you would like to try a certain role. Take on that role today and contribute from there’. So if they are a bit reserved or a bit hesitant, they can take on that other role and ask questions. I challenge them to do that, because the study group would benefit from it. (P11)*

Participants explained that the more they knew about their students, the more effectively they could stimulate engagement. Participants gained insight into the engagement requirements of their students, as well as cues indicating their disengagement, including students’ expressions and reactions. This enabled them to implement strategies to re-engage students in such situations.

#### Course design elements

Participants reported knowing the course design very well. They knew the objectives, assignments, roles, methods, activities, and assessment. Although some parts of the course design were non-negotiable boundaries, participants took the initiative to choose and adapt their approach wherever possible, to optimally stimulate student engagement. Participants often mentioned that in the first sessions with a new study group, not enough time was dedicated to getting to know the students. They used their experience to make changes to the given schedule and assignments and created time for what they found important.

*As a teacher you should be able to think beyond the rules and the specifics of one assignment and reflect on the purpose of the sessions and the course itself. The purpose is not to brainstorm a certain number of cases in a given time, or to follow a certain method to the letter. […] The purpose is that students learn to think in a certain way, and you should focus on that. (P2)*

### Facilitating the active learning process

#### Observing

Participants commented on the importance of observing the students to regulate their engagement. They described observing as the active perception of what is happening in the moment. It involved recognizing and understanding subtle signals and behaviors. The teachers said they always did something, because at the very least they were observing.

*I am always observing. In the beginning I aim to understand the dynamics of the groups and the roles of each of the students. Just to get to know them […] To understand what kind of a group they are and how they collaborate. I am looking for indications of how the learning process unfolds and if they are making progress. […] I look for who is contributing and who is not. (P8)*

Observing was described as a complex competency: during study group sessions, multiple things would usually occur simultaneously and quickly. Participants noted that their students’ engagement declined when they were distracted by something that reduced their ability to observe. For example, when participants were overly involved with the content, they would miss the nonverbal cues of students or fail to notice private conversations among them. They would then miss the opportunity to intervene.

#### Analyzing

Participants described analyzing as the step in facilitating the active learning process in which they made assessments or interpretations of their observations: does what they observe deviate from their expectations? If so, what could it mean? Participants described how this step was important before making a decision, because they could think about their observation from different perspectives, for example, their aim (what might this observation mean in light of the psychological safety I hope to provide?) or approach (what do I know about this student, and how might that affect their behavior?). After this consideration, participants would realize that there were a number of options they could choose from, with different outcomes.

*So my idea is that at least you become aware of options A, B, and C. […] And if you feel doubt about what to do, then you can dive into that doubt. Trying to feel what that doubt is, right? And then, well, then you have a bit more clarity regarding which choice you want to make, and why. So then you can justify it better for yourself. (P3)*

#### Deciding

Participants described deciding on a course of action as the final step in facilitating the active learning process, after which a new process began with observing the effects of their actions. Reflecting on their development, participants noted that they used to frequently experience tensions between various possible courses of action, complicating their decision-making process. One participant explained how she dealt with the tension between ‘doing the assignments and complying with the course manual’ and ‘creating personal opportunities for student development’ by adhering to her educational value of ‘personal development’:

*You have that tension. But only when you forget that they are human beings, and they are in a process of developing themselves. And that they all have something different to learn from the study group sessions, not necessarily only the course’s learning objectives. […] Of course, the course learning objectives, they need to learn those for their exams. But the study group sessions are also about gaining confidence and daring to speak in front of an audience, daring to voice your opinion, realizing the effects of always being late on fellow students and receiving comments about that behavior. I believe those experiences develop them as human beings. (P2)*

## Discussion

**Figure 1:**
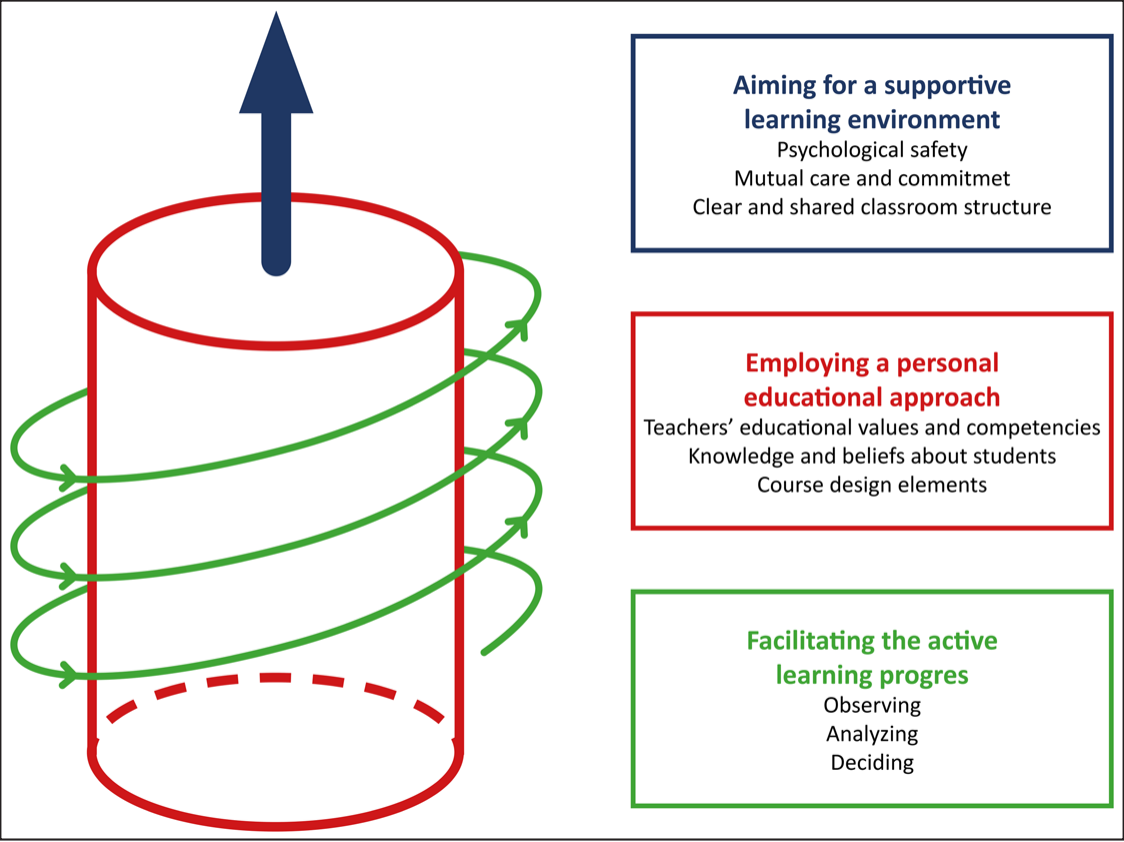
Grounded Theory of how expert teachers stimulate high levels of student engagement. The blue arrow illustrates how expert teachers cultivate an increasingly supportive learning environment through their personal educational approach. As this process unfolds, they observe their students, analyze cues related to their aims and approach, and decide on a course of action.

In this study, we examined how expert teachers stimulated high levels of student engagement in small-group active learning. The theory we have constructed emphasizes three aspects. First, there was consensus among expert teachers on the importance of a supportive learning environment and the ability to facilitate an active learning process. Second, the expert teachers in this study described how they had developed and employed a personal educational approach, recognizing the contextual nature of student engagement. Third, student engagement was viewed as an integrated process consisting of all elements of the constructed theory. High levels of student engagement required extensive competencies in all the identified elements. Besides stimulating high levels of student engagement, participants reported that their competencies and practices prevented truly disruptive student behaviors in class. Figure 1 visualizes how the three components of the theory jointly stimulated student engagement.

Our findings contribute to the discussion about the paradox between the effectiveness of faculty development initiatives and the continuous challenge of student engagement in medical education [15,21,27,28]. First, the theory we constructed identified which knowledge, skills, and attitudes were essential for the expert teachers. Currently, faculty development in medical education is commonly short in duration (e.g. single workshops) and limited in scope (e.g. interactive techniques like questioning) [25,27]. Acknowledging these limitations, it is apparent that while faculty development initiatives do enhance teacher competencies in student engagement, they may not fully encompass all the essential aspects of success as reported by the participants in this study. This observation is not to diminish the value of these initiatives, but to underscore the need for a more comprehensive approach that integrates all reported aspects. Second, building on the previous point, our findings indicate that student engagement is context-dependent, as shown by the three aspects of ‘personal educational approach.’ All participating expert teachers agreed that there is no one-size-fits-all method to engaging students. Although they reported that they had learned general strategies for stimulating student engagement through faculty development initiatives, the expert teachers had to figure out which to use and how to make them work. Consequently, teachers enrolled in faculty development initiatives could, and that is what the expert teachers in this study did, consider the question ‘which approach might be effective in this context, taking into account my own set of values and competencies, the characteristics of my students, and the specifics of the course I am involved in?’ Moreover, through the process of observing, analyzing, and deciding on a course of action, the expert teachers remained reflective on the impact of their approach and could adapt if needed. In conclusion, while faculty development serves as a cornerstone for developing teachers’ competencies in stimulating student engagement, our research highlights the importance of a comprehensive and contextualized approach to ensure a positive impact on actual teaching practices.

### Limitations and strengths

Although this study provides useful insights for faculty development, there are several issues to consider when interpreting the results. The selected expert teachers were medical teachers from one Dutch university in a course employing a case-based (years 1 and 2) and team-based (year 3) learning approach. Thus, the sample selection, geographic context, and teaching method may have influenced our findings. Additionally, the teachers’ educational values in this study aligned well with active learning. Future research could explore whether such an alignment is a key factor for successful active learning implementation. Lastly, we based our grounded theory on teacher interviews and used a limited ‘theoretical sampling’ procedure. An extended theoretical sampling procedure in which other methods (like classroom observations or student interviews) are integrated could further advance our understanding.

The main strength of this study lies in the application of appreciative inquiry. This method has been identified as an ‘exciting potential’ for medical education research due to its focus on ‘what is going well’ and its generative process [36]. We experienced the interviews to be characterized by high positive energy and rich information. Participants spoke openly about their experiences and beliefs and often indicated feeling inspired and having learned something about themselves.

Finally, we want to consider the inclusion of teachers with varying levels of expertise in this study. Although all participants met our inclusion criteria, some had more experience or qualifications than others. While this could be seen as a limitation, as it may influence the findings of our study, we argue that it was a strength. For example, during the interviews, all participants expressed that they value psychological safety. However, some were hesitant in describing how they achieved it, while others had developed comprehensive approaches they could articulate. This variation reinforces firstly the importance of psychological safety, and secondly the implication for faculty development for a comprehensive and contextualized approach, allowing teachers of varying levels of expertise to develop their competencies in engaging students.

## Conclusions

In conclusion, this study explored how expert teachers engaged their students in small-group active learning sessions. Our constructed theory described student engagement as an integrated process consisting of three components, which demanded extensive competencies from teachers in each component: 1) aiming for a supportive learning environment; 2) employing a personal educational approach; and 3) facilitating the active learning process. Although there was consensus about the required competencies, participants recognized the contextualized nature of student engagement. These findings highlight the need for faculty development initiatives, which aim to prepare medical teachers to teach in small-group active learning settings, to adopt a more encompassing, context-sensitive approach that considers the complexity of student engagement. Furthermore, the findings could encourage teachers to adopt a reflective mindset that enables them to adapt general strategies to strategies tailored to them in their context.

## Additional File

The additional file for this article can be found as follows:

10.5334/pme.1245.s1 Supplementary Materials. Interview guide.
