# Supplementary material for: Learning from the Experts: Stimulating Student Engagement in Small-group Active Learning
Source: Perspect Med Educ. 2024 Apr 15;13(1):229–38. doi: 10.5334/pme.1245 (PMC11025576; doi:10.5334/pme.1245)
Supplement: Supplementary Materials. — Interview guide. [file pme-13-1-1245-s1.pdf]

Supplementary materials

Interview guide

Structure of interview	Questions
Discover question<br><br>*(Goal: focus on positive teaching experience and collaboratively discovering what underlying processes might have contributed to that experience)*	Can you tell me a story about a recent study group meeting where everyone was active and engaged with each other and with a study assignment? So really a positive experience. This can be a moment, or a group, or a specific study assignment that you were impressed with or proud of.<br><br>Example follow-up questions:<br>- In that situation, what was your role? What did you do, what did you think, and what did you feel?<br>- What is it about this story that makes it such a positive experience for you?<br>- What do you think is necessary for such a wonderful thing to happen?<br>- What can we learn from this story, in your opinion?
Contrasting question<br>*(Goal: gain more insight into underlying processes by exploring student*	With the same study group as the first story, or maybe another group, does it sometimes not work out?<br><br>Example follow-up questions:

engagement from an opposing perspective)	- Can you tell me more about that, just like with the positive teaching experience just now, or describe what it was like?<br>- In that situation, what did you do, what did you think, and what did you feel?<br>- What is it about this story that makes it difficult for you?<br>- And as before, what do you think contributed to this happening?<br>- What have you learned from this situation?
Broadening question *(Goal: gain more insight into underlying processes by exploring a different story)*	Can you share another such positive experience with a study group that might have been very different from the story you just told?<br><br>Example follow-up questions:<br>- What makes this experience also positive for you but still different?<br>- Again, what was your role? What did you do, what did you think, and what did you feel?<br>- What do you think was necessary for this wonderful thing to have happened? Are they the same things you just mentioned, or was there something else at play?<br>- What can we learn from this story, in your opinion? Is it a confirmation of the above, or is there something else?
Dream question	If I ask you to dream about active learning and students

(Goal: reflecting if and how the tutor training supported first time tutors)	engaging in the learning process...<br>- What are your dreams? What do they look like? Can you describe that?<br><br>Example follow-up questions:<br>- Imagine we are living in the future, so two years from now, for example, and your dreams have come true! And indeed it is the case that… (mention dreams)<br>i. What has changed in the next two years that has made this possible? (in course design, teachers, students, ...)<br>ii. How has this change been able to happen – who has done what?<br>iii. What has changed about you in those two years? Have you developed something?<br>iv. What makes that aspect so important that you are paying attention to it?
Closing questions<br>*(Goal: collaboratively reflect on the interview and distill key points)*	Example question:<br>-What do you think have been key points in this interview?<br>- Which aspects of everything we discussed do you think are essential for the engagement of the students in your study groups?<br>- What would you like to pass on to beginning tutors if they want to learn how to engage their students?

	- Do you have anything to add to what we have discussed and what may be important for this research?<br>- What have you yourself learned from this interview?
